# Therapeutic Potential of Probiotics in the Modulation of Antibiotic Resistance in *Helicobacter pylori*

**DOI:** 10.3390/biomedicines14051044

**Published:** 2026-05-04

**Authors:** Lazzat Zhussupbekova, Alina Bulatova, Dinara Nurkina, Klara Kurmangaliyeva, Venera Rakhmetova, Aigyul Izhanova, Kaergeldy Makhambetov, Elmira Akhmedyarova

**Affiliations:** 1 Department of Internal Diseases, Astana Medical University, Astana 010000, Kazakhstan; zhussupbekova.l@amu.kz (L.Z.); kurmangalieva.k@amu.kz (K.K.); rahmetova.v@amu.kz (V.R.); makhambetov.k@amu.kz (K.M.); akhmedyarova.e@amu.kz (E.A.); 2 Department of Internal Medicine, Asfendiyarov Kazakh National Medical University Kazakhstan, Tole bi Street 94, Almaty 050000, Kazakhstan; aygul-izhanova@mail.ru

**Keywords:** *Helicobacter pylori*, antibiotic resistance, probiotics, intestinal microbiota, eradication therapy, dysbacteriosis, *Lactobacillus*, *Saccharomyces boulardii*

## Abstract

Over the past decade, the growth of *Helicobacter pylori* antibiotic resistance has had an increasingly significant impact on the choice of eradication therapy regimens, significantly limiting the number of effective treatment options. The Maastricht VI guidelines consider the use of probiotics as one way to optimise therapy and increase the likelihood of successful eradication. In this regard, the study of the possible mechanisms of action of probiotic preparations on antibiotic-resistant strains of *H. pylori*, as well as their possible role in preventing the development of resistance, is of considerable interest and may contribute to improving the quality of eradication therapy in the near future. The aim of the study is to determine the role of probiotics in modifying the microbiota during and after eradication therapy, as well as to assess their potential impact on the development of antibiotic resistance. A search for scientific publications was conducted in international and national bibliographic databases: PubMed, Embase, Scopus, Web of Science Core Collection, Google Scholar, and eLIBRARY.ru. The search was conducted in English, Russian, and Kazakh for the period from 26 April 2015 to 14 July 2025. The review includes 41 publications. Eradication therapy has a pronounced negative effect on the intestinal microbiota, leading to disruption of its composition and the development of side effects that reduce treatment tolerability. A number of studies in recent years have demonstrated a link between antibiotic therapy and the development of antibiotic resistance caused by genetic rearrangements and mutations in the gut microbiota genome. As an additional approach to correcting these adverse effects, special attention is paid to the use of probiotics. According to the research results, probiotic preparations help reduce the frequency of side effects of eradication therapy and may increase its effectiveness against antibiotic-resistant strains of *H. pylori*. The use of probiotic preparations in the treatment of resistant strains of *Helicobacter pylori* is a promising direction that opens up new opportunities for optimising eradication regimens. However, this approach requires further clinical and experimental studies to confirm its effectiveness and form reasonable prognostic conclusions.

## 1. Introduction

Since 2017, the World Health Organisation has included *Helicobacter pylori* in the list of bacteria posing the greatest threat due to the growth of antibiotic resistance, which reinforces the importance of the rational use of antibacterial drugs [1]. According to research, resistance to clarithromycin currently reaches 30% in Southern European countries and up to 50% in China [2]. The decrease in the sensitivity of *H. pylori* to antibiotics leads to a decrease in the effectiveness of standard eradication therapy, and the increase in the frequency of therapeutic failures is accompanied by an increased risk of complications, including peptic ulcer disease and stomach cancer. This problem is exacerbated by the limited range of effective antibacterial agents, as well as the widespread and often suboptimal use of antibiotics in clinical practice [3].

Over the past decade, a number of studies have been conducted on the dynamics of *H. pylori* resistance to key therapeutic drugs, including clarithromycin, amoxicillin, levofloxacin, and metronidazole. According to WHO data, resistance to clarithromycin in Southeast Asian countries increased from 13% in 2006–2008 to 21% in 2012–2016, while resistance to levofloxacin in the Western Pacific region increased from 12% to 31% over the same period [4]. Of particular concern is the increase in multidrug resistance of *H. pylori*, which in some regions reaches 48.2% in China and 42.9% in Korea [5]. These data confirm the need to search for new approaches to improve the effectiveness of eradication therapy.

The relevance of this study is determined by the continuing threat of reduced effectiveness of standard *H. pylori* treatment regimens, which requires the investigation of additional methods capable of improving treatment outcomes. According to a large systematic review and network meta-analysis in 2024, the inclusion of probiotics in the eradication regimen significantly increases the frequency of successful eradication compared to antibiotic monotherapy [6]. It has also been shown that the use of probiotics after standard quadruple therapy reduces the frequency of infection recurrence and decreases the severity of gastrointestinal side effects [7]. These data are consistent with the results of a 2024 meta-analysis, which demonstrated a significant reduction in the risk of adverse reactions during antibiotic therapy when probiotics were used (RR = 0.54), which may have a beneficial effect on treatment tolerability [8]. Current data confirm that probiotics are a promising addition to eradication therapy, potentially capable of improving clinical outcomes and limiting the further growth of antibiotic resistance.

The aim of this study was to determine the role of probiotics in modifying the microbiota during and after eradication therapy, as well as to assess their potential impact on the development of antibiotic resistance.

## 2. Search Strategy and Scope

A search for scientific publications was conducted in international and national bibliographic databases: PubMed, Embase, Scopus, Web of Science Core Collection, Google Scholar, and eLIBRARY.ru in English, Russian, and Kazakh. The search time interval covered the period from 26 April 2015 to 14 July 2025, which corresponds to the stage between the publications of Maastricht V (2015), which focuses on the problem of *H. pylori* antibiotic resistance, and Maastricht VI (2022), which discusses modern approaches to the use of probiotics in eradication regimens. Keywords and MeSH terms were used for the search, including: “*Helicobacter pylori*”, “antibiotic resistance”, “probiotics”, “gut microbiota”, “treatment failure”, “microbiota modulation”, “eradication efficacy”, “dysbiosis”, “*Lactobacillus*”. Combinations were formed using the logical operators AND and OR.

The search results identified 432 publications, of which 41 met the inclusion criteria and were included in the final review. The inclusion criteria were randomised clinical trials, cohort studies, meta-analyses, and systematic reviews containing data on patients over 18 years of age with confirmed *H. pylori* infection who received standard eradication therapy. Exclusion criteria included the following: narrative reviews without original data, studies involving children, animals, duplicate publications, and works not available in full-text format. Publications were selected sequentially: scanning of titles and abstracts, followed by analysis of full texts according to the criteria. Data on the types of interventions, sample characteristics, analysis methods, and main clinical outcomes were extracted from the included studies.

## 3. The Effect of *Helicobacter pylori* Eradication Therapy on the Composition, Diversity, and Functional Activity of the Intestinal Microbiota

*Helicobacter pylori* eradication therapy has a pronounced effect on the intestinal microbiota, mainly in the early post-treatment period. Most clinical studies indicate a rapid but largely reversible disruption of the taxonomic structure of the gut microbiota and a decrease in its alpha diversity (Table 1).

In the early post-treatment period, a decrease in alpha diversity and significant shifts in the main types are recorded: there is a decrease in the proportion of *Bacteroidetes* and *Actinobacteria* with a simultaneous relative increase in representatives of *Proteobacteria* and some conditionally pathogenic genera (e.g., *Escherichia*/*Enterobacteriaceae*). Thus, after eradication, the authors recorded a decrease in the proportion of *Bacteroidetes* (on average, by 15% by the 12th month relative to the baseline value) and variable dynamics of *Proteobacteria*: 3.7% (6 months), 6.8% (12 months), and 8.4% (18 months) at the observation points. These changes were accompanied by an increase in the proportion of SCFA-producing genera by 6 months [9] (Table 1).

A number of small randomised clinical trials involving a total of over 130 patients have shown that a marked shift in the taxonomic profile of the gut microbiota occurs as early as 1–2 weeks after the start of eradication therapy. Thus, the relative proportion of the *Proteobacteria* phylum increased from baseline values of around 9–10% to 50–60%, whilst the proportion of *Bacteroidetes* decreased from 20 to 25% to <1% (approximately 0.5%). The recovery of these indicators to values close to baseline occurred gradually and, as a rule, was only complete after 1 year. These changes were accompanied by a statistically significant decrease in alpha diversity, as measured by the Chao1 index, in the second week of therapy compared with baseline (*p* = 0.006) [10,15,16] (Table 1).

The results obtained are confirmed by a larger randomised clinical trial in China in 2024, involving more than 520 patients, which also recorded a significant decrease in the alpha diversity of the intestinal microbiota according to the Chao1 index after completion of eradication therapy (*p* = 0.014) (Table 1). Despite differences in treatment regimens and study design, the short-term reduction in microbiota diversity and the dominance of *Proteobacteria* are an effect of the antibacterial action [12].

At the same time, data from studies with a longer observation period indicate a gradual recovery of the microbiota. In three RCTs with repeated sampling 8–48 weeks after therapy, the proportion of *Proteobacteria* decreased from peak values of about 58% to 15–20%, while the proportion of *Bacteroidetes* increased from 0.5% to 18–22%, approaching baseline values [11,13] (Table 1). Taken together, these data indicate the high plasticity of the gut microbiota and its ability to self-repair.

A large multicentre randomised study by Cong He et al., involving 276 people, demonstrated marked changes in the composition of the intestinal microbiota after 14 days of bismuth-containing quadruple therapy, characterised by a predominance of *Proteobacteria* and a simultaneous decrease in *Firmicutes* and *Bacteroidetes* [14] (Table 1).

A common and clinically significant finding in most studies is the severity of dysbiotic changes in the intestinal microbiota in the early post-treatment period, accompanied by a decrease in alpha diversity and a disruption in the proportional relationship between the main bacterial taxa. These changes are associated with the development of side effects of therapy, such as diarrhoea and other dyspeptic disorders. At the same time, most studies demonstrate the temporary nature of the identified disorders, with a tendency to restore the composition of the intestinal microbiota within approximately 1 year after the end of therapy. The combined data from randomised clinical trials and meta-analyses indicate that the main effect of *H. pylori* eradication therapy is not a quantitative depletion of the intestinal microbiota but a proportional change in the composition of its components, which highlights the relevance of finding strategies aimed at accelerating the restoration of the microbiota and improving the tolerability of treatment. Thus, the presented data on changes in the composition of the intestinal microbiome can be considered as one of the mechanisms underlying predisposition to antibiotic resistance.

## 4. The Link Between *H. pylori* Eradication Therapy and the Selection of Resistant Strains of Intestinal Microbiota

The use of antibacterial drugs, as discussed in the previous section, is one of the key factors affecting the structure and functional state of the intestinal microbiota, which allows us to consider changes in the microbial community as a potential mechanism for the formation of antibiotic resistance. A number of studies have demonstrated a link between *H. pylori* eradication therapy and a transient increase in the resistance of opportunistic intestinal microorganisms to antibacterial drugs.

This correlation was demonstrated in a large randomised clinical trial in Taiwan in 2019, involving more than 1200 patients. Before the start of therapy, no statistically significant differences in the prevalence of antibiotic resistance were found. However, by the second week of treatment, patients receiving combination eradication therapy showed a sharp increase in the growth of resistant *Escherichia coli* and *Klebsiella pneumoniae* to ampicillin (from 12% to 66%), cefazolin (from 13% to 43%), and levofloxacin (from 8% to 34%). At the same time, at week 8 and 1 year after the end of therapy, no significant differences from the baseline level of antibiotic resistance were detected (*p* > 0.07). At the same time, a marked decrease in the alpha diversity of the intestinal microbiota was observed after 2 weeks of therapy (*p* = 0.0002), followed by recovery by week 8 (*p* = 0.14) and 1 year (*p* = 0.81). Thus, the peak of antibiotic resistance coincided with the maximum decrease in alpha diversity of the microbiota, suggesting a correlation between the two [13].

Additional data on possible mechanisms of resistance formation were obtained by analysing the expression of antibiotic resistance genes (ARGs). A randomised clinical trial conducted in Hong Kong involving more than 40 patients showed that the relative abundance of macrolide–lincosamide–streptogramin (MLS) antibiotic resistance genes increased significantly at week 6 after the start of therapy (*p* = 0.03), followed by a return to baseline levels after 6 months. A similar trend was observed for fluoroquinolone resistance genes (*p* = 0.03) and multidrug resistance genes (*p* = 0.01) [17]. A study by Zhao Meiqi et al. found that representatives of the genera *Escherichia* and *Klebsiella* are the main bacterial representatives of highly mobile ARGs. Prior to antibiotic treatment, the proportion of ARGs in metagenomes was 17.7%, whereas after combined eradication therapy, this figure increased to 46.43%. At the same time, there was a significant increase in the proportion of mobile genetic elements in DNA sequences containing ARG, from 4.85% to 14.58%. These data suggest the involvement of horizontal gene transfer in the observed increase in the level of antibiotic resistance in the intestinal microbiome [18].

**Figure 1:**
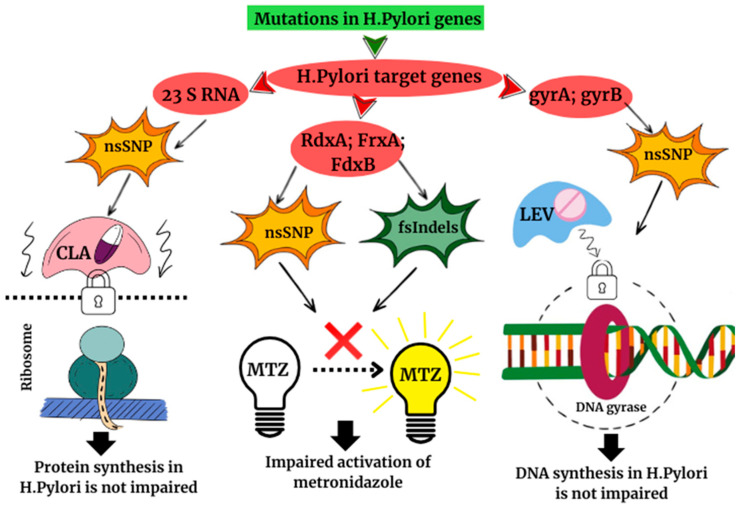
The mechanism underlying the development of acquired antibiotic resistance in *H. pylori*. The acquisition of antibiotic resistance in *H. pylori* is mainly due to chromosomal point mutations. Mutations in clarithromycin (23S rRNA) targets interfere with the binding of the antibiotic to the 50S subunit, preserving protein synthesis; changes in rdxA, frxA, and fdxB disrupt metronidazole prodrug activation, rendering it ineffective; mutations in gyrA and gyrB deprive levofloxacin of its ability to inhibit DNA gyrase, allowing H. pylori DNA replication to continue despite the presence of an antibiotic. Abbreviations: nsSNP, single nucleotide polymorphism; fsIndels, insertion/deletion of nucleotides; CLA, Clarithromycin; MTZ, Metronidazole; LEV, Levofloxacin.

On the other hand, the development of resistance may be due to changes in the *H. pylori* genome. A study involving 112 patients in Shanghai in 2022 identified key genes whose mutations are associated with the development of resistance to major antibacterial drugs. These include the 23S rRNA, gyrA, gyrB, rdxA, frxA, and fdx genes. Point mutations, realised through non-synonymous single nucleotide polymorphisms (nsSNPs) and insertions/deletions (fsIndels), lead to the development of resistance to clarithromycin, levofloxacin, and metronidazole (Figure 1) [19].

Thus, modern studies consider the development of antibiotic resistance in the context of *H. pylori* eradication therapy as a multifactorial process involving a transient decrease in the alpha diversity of the intestinal microbiota, selective enrichment of the intestinal microbiome with antibiotic resistance genes, potential involvement of horizontal gene transfer mechanisms, and accumulation of mutations in the pathogen genome (Figure 1). The widespread use of antibacterial drugs, repeated courses of therapy when treatment is ineffective, and the unjustified prescription of antibiotics contribute to microbiome and genetic changes, creating conditions for the further spread of antibiotic resistance. When considering the processes occurring during eradication therapy as a whole—from alterations in the compositions of the intestinal microbiota to genomic rearrangements—our understanding of the potential mechanisms underlying the development of this complication is broadened. This problem requires a comprehensive approach to its study and the development of strategies for rational antimicrobial therapy.

## 5. The Effect of Probiotics on the Modulation of the Intestinal Microbiota During and After *Helicobacter pylori* Eradication Therapy

Based on the problems described above, the search for additional approaches to optimise *H. pylori* antibiotic therapy, as well as to improve the quality of life of patients during and after treatment, remains a pressing issue in modern gastroenterology. With the publication of the Maastricht VI (2022) guidelines, the use of probiotics has attracted attention not only as a means of reducing the incidence of side effects but also as a potential factor in increasing the effectiveness of eradication therapy.

According to the Maastricht VI guidelines, the use of probiotics is associated with an increase in the effectiveness of *H. pylori* eradication therapy. This finding is confirmed by a meta-analysis of 40 studies, in which the probiotic support group had a higher eradication rate compared to the control group (*p* < 0.001) [20]. Comparable results are presented in two meta-analyses covering 19 and 25 studies, respectively, where the addition of probiotics was accompanied by an increase in the eradication rate compared to classic antibiotic therapy (*p* = 0.0004) with no statistically significant heterogeneity (I^2^ = 0%) [21,22].

Further evidence was obtained from two large randomised clinical trials conducted in Greece and China, involving a total of around 1000 participants, in which the eradication rate in the probiotic group was statistically significantly higher than in the control group (92.0% vs. 86.8%; *p* = 0.028) [23], and a reduction in the incidence of infection recurrence was also noted (9.2% vs. 19.2%; *p* = 0.021) [7]. Taken together, these results indicate the advisability of including probiotics in combination therapy to enhance the efficacy of standard *H. pylori* eradication regimens.

One of the key benefits of probiotics when used in combination with eradication therapy is a reduction in the incidence of side effects associated with antibiotic treatment. The most common adverse reactions are gastrointestinal disorders, which account for the primary protective effect of probiotics. In a large randomised clinical trial by Zhao Jie et al. in 2025, antibiotic-associated diarrhoea was recorded in 7.5% of patients receiving probiotics, compared with 18.3% in the control group (*p* = 0.013) [7]. These findings are supported by the results of two meta-analyses, comprising over 40 studies, in which the combined use of probiotics and antibiotics significantly reduced the risk of diarrhoea (*p* < 0.00001) and nausea (*p* = 0.02) [21,22].

In addition, probiotic supplementation has been associated with a reduction in abdominal pain and flatulence. In a small randomised clinical trial in 2022 involving 80 patients, a reduction in abdominal pain was observed in 42% of patients in the probiotic group compared to 19% in the control group (*p* < 0.001), and a reduction in bloating in 25% and 17%, respectively (*p* < 0.001) [24].

At the same time, a number of studies demonstrate the limited effect of probiotics on the quantitative and qualitative composition of the intestinal microbiota. For example, a meta-analysis of 30 studies showed that the relative content of the main taxa of the intestinal microbiota associated with probiotics did not undergo statistically significant changes before and after eradication therapy. In particular, changes in the abundance of *Lactobacillus*, *Bifidobacterium*, *Bacteroides*, and *Enterococcus* did not reach statistical significance [25] (Table 2). Similarly, two large randomised clinical trials in China in 2022 and 2020, with a total of more than 400 participants, did not confirm the ability of probiotics to increase or maintain α-diversity of the intestinal microbiota after *H. pylori* eradication [14,15] (Table 2).

Probiotic support as part of *H. pylori* eradication therapy is associated with a moderate increase in treatment efficacy and a significant reduction in the frequency of side effects. At the same time, the severity of the clinical effect depends on the probiotic strains used and the antibiotic therapy regimens. Despite the proven effect of probiotics on reducing gastrointestinal symptoms, their role in restoring and maintaining microbial diversity, including α-diversity, remains a subject of debate. The published data on the restoration of taxonomic diversity of the gut microbiome did not confirm its clinical and statistical significance in our study, leaving open the question of the presence of such a property among probiotics. The heterogeneity of the available data highlights the need for further research to clarify the clinical significance of probiotics in the correction of antibiotic-induced intestinal dysbiosis.

## 6. The Potential of Probiotic *Lactobacillus* Strains in Reducing the Prevalence of Antibiotic-Resistant Strains of *Helicobacter pylori*

Based on the results of the previously included studies, it can be concluded that one of the most significant complications of *H. pylori* eradication therapy at the present stage is the development of antibiotic resistance, which significantly limits the choice of effective antibacterial drugs. Given the limited list of antibiotics used in eradication regimens, the growth of pathogen resistance poses a serious clinical problem, especially in the context of frequent relapses of infection.

Data on the efficiency of probiotics, specifically the *Lactobacillus* strains, against antibiotic-resistant strains of *H. pylori*, are currently based primarily on the results of preclinical experimental studies. In an in vitro model, *Lactobacillus* pentosus LPS16 and its culture medium (MRS) were used to evaluate the inhibitory potential of probiotics. Thirty-five strains of *H. pylori*, including 18 strains resistant to at least one antibacterial drug, were studied in a diffusion assay. Under the influence of LPS16, the viability of *H. pylori* decreased from 10^8^ to 10^1^ colony-forming units, with statistically significantly larger inhibition zones (*p* < 0.01). The data obtained indicate that the antimicrobial effect is due not only to a decrease in pH but also to the specific bactericidal action of metabolites, including lactic acid [26].

Similar results were obtained in studies using *Lactobacillus delbrueckii* subsp. *bulgaricus* (GLB) strains, which evaluated the antimicrobial activity of probiotics against 18 strains of *H. pylori*, 11 of which were antibiotic-resistant. Cell-free supernatants (CFSs) of two GLB strains inhibited the growth of more than 81% of the tested strains, while CFS of four GLB strains were active against 71.4–87.5% of antibiotic-resistant *H. pylori* isolates [27].

In a study with a similar design, which was aimed at evaluating the effect of probiotics on eight strains of *H. pylori* resistant to more than one antibiotic, various strains of *Lactobacillus* and their cell-free supernatants were used. According to the results obtained, CFS *L. acidophilus*, *L. rhamnosus, L. reuteri*, and *L. casei* inhibited the growth of all eight strains of *H. pylori*, while L. fermentum and L. plantarum were active against four and six strains, respectively. At the same time, the antimicrobial effect was significantly higher when using CFS compared to live bacterial cultures: the reduction in bacterial load when using CFS ranged from 1 to 6 log, while when using live cultures it did not exceed 1 log [28]. These data indicate that there is no consensus on the dominant mechanism of the antagonistic action of probiotics—the direct effect of live microorganisms or the indirect effect of their metabolites.

Thus, the experimental data presented indicate the inhibitory effect of a number of probiotic strains on *H. pylori*, including antibiotic-resistant isolates. The most frequently studied representatives of the *Lactobacillus* genus have demonstrated potential efficacy both when used alone and in combination with antibiotic therapy, depending on the strain used [29]. Despite the predominantly preclinical nature of the available data, the results obtained indicate the promise of further study of *Lactobacillus* strain probiotics as an adjunctive tool in the strategy to overcome *H. pylori* antibiotic resistance. However, it is worth noting that despite the widespread use of this probiotic in clinical practice to facilitate therapy, the evidence for its effects in relation to resistant forms of *H. pylori* is severely limited by the lack of clinical studies in humans. At the moment, the results presented in studies of an in vitro model cannot confirm the presence of the intended effect in therapeutic practice.

## 7. Advantages of Using *Saccharomyces boulardii* in the Treatment of *H. pylori*

In the Maastricht VI recommendations, among probiotic supplements that have a positive effect in eradication therapy, in addition to *Lactobacillus* strains, special attention is paid to the use of *Saccharomyces boulardii* yeast. In addition to reducing the incidence of side effects during antibiotic therapy, it increases its effectiveness through a direct inhibitory effect on *H. pylori*. However, this mechanism of suppression, represented by the production of antimicrobial substances and competition with *H. pylori* in colonising the mucous membrane, is still questioned and requires further study.

The main advantage of *S. boulardii* is the suppression of side effects, which, according to Maastricht VI, may be the main reason for the increased frequency of *H. pylori* eradication, prevailing over the direct effect on the pathogen. Thus, the effect of *S. boulardii* on the development of adverse effects is observed in four meta-analyses [30,31,32,33], according to which the use of the drug reduced the frequency of common side effects, in particular, diarrhoea, bloating, constipation and nausea [31]. These results are confirmed by three randomised clinical trials involving a total of more than 450 people conducted in China, Ecuador, and Iran, according to which the incidence of side effects in groups of patients taking probiotics was significantly lower than in control groups (*p* < 0.05) [34,35,36].

**Figure 2:**
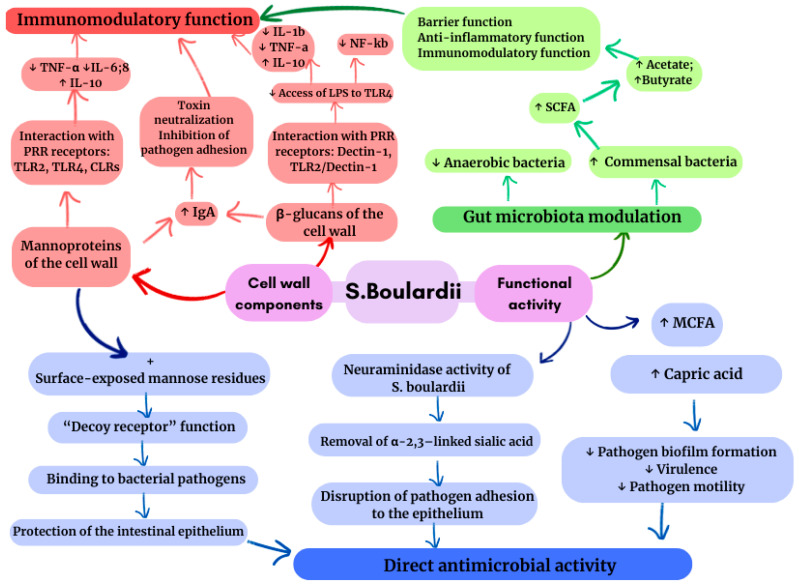
Mechanism of action of the probiotic *S. boulardii* in the gut. *S. boulardii cell* wall mannoproteins and β-glucans induce a controlled immune response by activating PRRs on enterocytes and macrophages with the release of cytokines (TNF-a; IL-6, 8, 10, 1b) without excess inflammation and stimulating IgA secretion. Taking *S. boulardii* increases the production of commensals of short-chain fatty acids (SCFA, including acetate, butyrate), which strengthens the barrier and anti-inflammatory function and modulates immunity. Mannoproteins act as “false receptors”, blocking the adhesion of pathogens; neuraminidase activity competes for binding to α-2,3-sial residues, inhibiting attachment; Medium-chain fatty acids (MCFAs) are released to reduce the activity, virulence, and biofilm formation of pathogens. Taken together, these mechanisms provide direct antimicrobial and immunomodulatory activity of *S. boulardii*. ↑ indicates an increase or upregulation, ↓ indicates a decrease or downregulation.

A change in the composition of the intestinal microbiota during treatment with *S. boulardii* is characterised by a decrease in the number of anaerobic bacteria (*Bacteroides*, *Clostridium*, etc.) with a simultaneous increase in the number of commensal bacteria [37]. This fraction of bacteria, including *Bifidobacterium*, *Lactobacillus*, etc., provides the synthesis of short-chain fatty acids (SCFAs) using components of the *S. boulardii* cell wall, such as glycans, mannoproteins, and chitin, as a substrate. The compounds that make up *S. boulardii* are responsible for its function as a postbiotic [38] (Figure 2). Acetate and butyrate, which are the main SCFAs in intestinal epithelial cells, participate in barrier, anti-inflammatory, and immunomodulatory functions. In turn, the mannoproteins that make up the cell wall of *S. boulardii* enable bacterial pathogens to adhere to yeast “false receptors” due to their connection to mannose residues on the surface, thereby protecting the intestinal epithelium from pathogenic invasion [39]. Along with preserving the epithelial barrier, *S. boulardii* participates in the production of saturated fatty acids, such as capric acid, which have antimicrobial properties that limit the activity and mobility of pathogens, also suppressing the formation of biofilms [38] (Figure 2).

The immunomodulatory capacity of *S. boulardii* is characterised by a shift in the cytokine profile: a decrease in pro-inflammatory cytokines (IL-8 and IL-1β) and an increase in anti-inflammatory cytokines (IL-4 and IL-10). It regulates the nuclear factor kappa B (NF-κB) and mitogen-activated protein kinase (MAPK) pathways, contributing to the anti-inflammatory mechanism [39]. In addition, *S. boulardii* increases IgA production, strengthening local mucosal defence and creating an effective immune barrier against *H. pylori* (Figure 2).

With regard to *H. pylori*, the results of studies indicate that the adhesion of this pathogen to intestinal epithelial cells is inhibited by the neuraminidase activity of *S. boulardii*, which is selective for α-2,3-linked sialic acid, a ligand for *H. pylori* binding, removing it. Thus, laboratory and clinical isolates of *H. pylori* treated with the probiotic showed a 41% and 31% reduction in adhesion, respectively (*p* < 0.05) [40], indicating the competitive effect of *S. boulardii* on the ability to attach to intestinal epithelial cells (Figure 2).

An argument in favour of the advantages of *S. boulardii* is its ability to influence the resistance of the intestinal microbiome after eradication therapy. According to the results of an original clinical study that examined ARG levels during *S. boulardii* treatment, a decrease in the content of genes providing resistance to lincosamides, tetracyclines, and MLS-B was observed in the group receiving the probiotic compared to the control group (edgeR, FDR < 0.05) [38]. A similar result can be observed in a study involving 68 patients, in which the resistance of *H. pylori* to clarithromycin, metronidazole, and levofloxacin was 91.3%, 100% and 60.9%, respectively. The eradication rate with *S. boulardii*, reaching 92.9%, suggests that a treatment regimen including this probiotic is effective regardless of antibiotic resistance [41].

The multifaceted mechanism of action of *S. boulardii* demonstrates its effect on treatment tolerance, maintenance of the intestinal epithelial barrier, control of inflammatory changes, preservation of intestinal microbiota diversity, and antibiotic resistance developing against the background of eradication therapy. The combination of beneficial effects of *S. boulardii* describes its advantages over the *Lactobacillus* strain, but a limited number of clinical studies cannot fully confirm the effectiveness of this probiotic. The difference in the level of research between *S. boulardii* and *Lactobacillus* makes the comparison insufficiently reliable. Thus, the inferiority of the overall picture still leaves room for further in-depth study of the possibilities of *S. boulardii* in treatment. Highlighting the main mechanisms underlying the development of antibiotic resistance and the emergence of a predisposition to it, we noted the following: changes in the composition of the intestinal microbiota and its genetic rearrangements. Thus, we begin our discussion of this problem with a search for the cause and consideration of ways to prevent it. The data presented today cannot fully answer the topical questions of the mechanism of the development of antibiotic resistance. However, based on the available information, we can assume new constructive theories.

## 8. Limitations

In the analysis of the data obtained, a number of studies presented the heterogeneity of the sample due to the fact that some studies used first-line eradication therapy [9,10,11,12,13,14,15,16,17,18,23,24,34,35,36,37,38], while others used the following lines after the initial ineffective treatment [17,41]. This limitation can be attributed to the leading correlation between the emergence of resistance and eradication therapy. Inadequate treatment regimens, namely triple therapy with clarithromycin, have been used in studies [9,11,34,35,36,37,38], which limited their relevance to current clinical guidelines (especially in regions with high clarithromycin resistance). This is difficult due to the geographical limitations of individual studies [7,9,10,12,13,14,16,17,18,19,23,24,27,28,34,35,36,37,38,41], the results of which may not apply to other populations with different levels of antibiotic resistance. The heterogeneity of the results obtained from publications may also be mediated by the presence of limitations such as the lack of dietary control among the study subjects [9,13,14,24,35,36,38], as well as the use of only one commercial probiotic with a fixed strain composition and dosage, which limits the generalizability of the results to other probiotics [14,15,16,23,24,26,27,28,34,35,36,38,41]. The use of in vitro studies [19,26,27,28,40] in comparison with the clinical results of eradication therapy is limited by the heterogeneity of laboratory strains and the multi-resistance of HP in patients.

In our study, the common limitations identified during the analysis of systematic reviews included heterogeneity of studies, heterogeneity in the quality of initial data, the use of different strains of probiotics, different schemes for the eradication of *H. pylori* (Table 1), and diagnostic control methods. Thus, the presented limitations in the interpretation and generalisation of the data do not allow us to speak unambiguously about the final results and general conclusions of the analysis but allow us to identify the focus of research and the main points for further in-depth study.

Taking into account the highlighted limitations of the study, as well as based on the objective data of the references used, it is possible to identify the main recommendations for further research in this area. It is necessary to expand the information boundaries of the work, primarily by conducting new randomised clinical trials using bismuth-containing quadtherapy in regions with high resistance to clarithromycin, as well as to increase the geographical representativeness of the data.

## 9. Conclusions

Having considered the therapeutic possibilities of probiotic supplements in combination with eradication therapy, the following confirmed aspects were highlighted: positive dynamics in increasing the effectiveness of the elimination of *Helicobacter pylori* and improving the tolerability of treatment by patients. At the same time, the evidence on the effects of probiotics on the composition and repair of the gut microbiota remains mixed, underscoring the need for further study. The data obtained on the advantage of *S. boulardii* require further research due to the limited sample size. Studies on the potential of *Lactobacillus* are not sufficiently informative for therapeutic practice, as they are exclusively preclinical in nature.

The potential role of probiotics in the prevention and modulation of the risk of developing multi-resistant strains of *Helicobacter pylori* can be considered as a prospect of innovative approaches to the development of optimal eradication regimens.

## Figures and Tables

**Table 1:** Influence of eradication therapy on microbiota.

Author	Country	Time	N	Therapy	Changes			
Theresa Wan-Chen Yap et al. (2016) [9]	Malaysia	June 2012– May 2014	17	Amoxicillin + Clarithromycin + PPI	*Bacteroidetes:* 52.09% (0 months) 47.82% (6 months) 36.84% (12 months) 38% (18 months)	*Firmicutes:* 32.91% (0 months) 37.82% (6 months) 43.53% (12 months) 38% (18 months)	*Actinobacteria:* 6.68% (0 months) 4.86% (6 months) 8.14% (12 months) 7.96% (18 months)	*Proteobacteria:* 5.77% (0 months) 3.69% (6 months) 6.75% (12 months) 8.4% (18 months)
Ping-I Hsu et al. (2018) [10]	Taiwan	August 2015– February 2017	11	Bismuth + PPI + Tetracycline + Metronidazole	*Bacteroidetes:* 24.3% (0 weeks) 0.53% (2 weeks) 9% (8 weeks) 19.61% (48 weeks)	*Firmicutes:* 45.3% (0 weeks) 36.73% (2 weeks) 55.61% (8 weeks) 49.72% (48 weeks)	*Actinobacteria:* 5% (0 weeks) 1.25% (2 weeks) 3.44% (8 weeks) 2.86% (48 weeks)	*Proteobacteria:* 9.9% (0 weeks) 57.75% (2 weeks) 24.10% (8 weeks) 16.39% (48 weeks)
Hidetaka Yanagi et al. (2017) [11]	Japan	April 2016– March 2017	20	Amoxicillin + Clarithromycin + PPI	*Bacteroidetes:* 0.9% (0 months) 4.5% (2 weeks) 4% (3 months)	*Firmicutes:* 69.9% (0 months) 71.8% (3 months)	*Actinobacteria:* 20.5% (0 months) 15.6% (2 weeks) 14.6% (3 months)	*Proteobacteria:* 7.2% (0 months) 0.9% (2 weeks)
Yi Hu et al. (2025) [12]	China	February 2023–January 2024	520	Vonoprazan + Amoxicillin	↓ Chao1 at 2 weeks (*p* = 0.014)	↓ *Firmicutes* *↑ Proteobacteria* *↓ Actinobacteria* (2 weeks)	At 8 weeks return to baseline values	
Jyh-Ming Liou et al. (2019) [13]	Taiwan	July 2015– April 2016	1214	Amoxicillin + Clarithromycin + PPI	↓ Alpha diversity at 2 weeks (*p* = 0.0002)	↓ *Firmicutes* *↑ Proteobacteria* *↓ Bacteroidetes* (2 weeks)	Recovery at 8 weeks (*p* = 0.14), and after one year (*p* = 0.81)	
Cong He et al. (2022) [14]	China	March 2019–November 2021	276	Esomeprazole + Bismuth + Amoxicillin + Furazolidone	↓ Chao1 (*p* < 0.05) at 2 weeks	↓ *Firmicutes* *↑ Proteobacteria* *↓ Bacteroidetes*	Recovery within a year	
Bo Tang et al. (2021) [15]	China	March 2019–November 2019	151	Esomeprazole + Bismuth + Amoxicillin + Furazolidone	↓ Chao1 (*p* < 0.05) at 2 weeks	↓ *Firmicutes* *↑ Proteobacteria* *↓ Bacteroidetes* (2 weeks)	At 4, 6, 8 weeks recovery to baseline values	

↑ indicates an increase or upregulation, ↓ indicates a decrease or downregulation. Abbreviations: PPI, proton pump inhibitors.

**Table 2:** Effects of probiotics on microbiota during *H. pylori* eradication.

Author	Country	Study Type	N	Therapy	Changes
Lijun Du et al. (2024) [25]	China	Systematic Review and Meta-Analysis	Thirty studies N = 1218	Clarithromycin-Based Triple Therapy + Probiotic	No differences were observed in the number of *Lactobacillus*, *Bifidobacterium*, *Bacteroides* and *Enterococcus* in patients before and after therapy
Bo Tang et al. (2021) [15]	China	Multicenter randomized controlled trial	N = 162	Esomeprazole, Amoxicillin, Furazolidone, Potassium Bismuth citrate + Medilac-S; *Enterococcus faecium* and *Bacillus subtilis*	The probiotic group showed similar beta-diversity values to the placebo group. Probiotic intake did not contribute to increasing or maintaining diversity after *H. Pylori* eradivation
Cong He et al. (2022) [14]	China	Multicenter randomized controlled double-blind placebo-controlled trial	N = 276	Esomeprazole, Bismuth, Amoxicillin, Furazolidone + *Bifidobacterium* Tetragenous Viable Bacteria Tablets	No significant differences were observed in Chao1 and Shannon indices between the probiotic and placebo groups. No statistical differences were observed between the groups

## Data Availability

Data sharing does not apply to this article, as no datasets were generated or analysed during the current study.

## Abbreviations

The following abbreviations are used in this manuscript:

SCFA	Short-chain fatty acids
RCT	Randomised controlled trial
ARG	Antibiotic resistance gene
MLS	Macrolide-lincosamide-streptogramin
MRS	de Man, Rogosa, and Sharpe (medium)
CFS	Cell-free supernatants
NF-κB	Nuclear factor kappa B
MAPK	Mitogen-activated protein kinase

## References

[1] Tacconelli E., Carrara E., Savoldi A., Harbarth S., Mendelson M., Monnet D.L., Pulcini C., Kahlmeter G., Kluytmans J., Carmeli Y. (2018). Discovery, research, and development of new antibiotics: The WHO priority list of antibiotic-resistant bacteria and tuberculosis. Lancet Infect. Dis..

[2] Fallone C.A., Moss S.F., Malfertheiner P. (2019). Reconciliation of Recent *Helicobacter pylori* Treatment Guidelines in a Time of Increasing Resistance to Antibiotics. Gastroenterology.

[3] Tshibangu-Kabamba E., Yamaoka Y. (2021). *Helicobacter pylori* infection and antibiotic resistance—From biology to clinical implications. Nat. Rev. Gastroenterol. Hepatol..

[4] Savoldi A., Carrara E., Graham D.Y., Conti M., Tacconelli E. (2018). Prevalence of Antibiotic Resistance in *Helicobacter pylori*: A Systematic Review and Meta-analysis in World Health Organization Regions. Gastroenterology.

[5] Lin Y., Shao Y., Yan J., Ye G. (2023). Antibiotic resistance in *Helicobacter pylori*: From potential biomolecular mechanisms to clinical practice. J. Clin. Lab. Anal..

[6] Tanashat M., Abuelazm M., Abouzid M., Al-Ajlouni Y.A., Ramadan A., Alsalah S., Sharaf A., Ayman D., Elharti H., Zhana S. (2025). Efficacy of probiotics regimens for *Helicobacter pylori* eradication: A systematic review, pairwise, and network meta-analysis of randomized controlled trials. Clin. Nutr. ESPEN.

[7] Zhao J., Chen J., Wang Y., Zhu C., Xia C., Yang W. (2025). The role of probiotic supplementation in reducing *Helicobacter pylori* recurrence after classic quadruple therapy. Front. Pharmacol..

[8] Yang Z., Zhou Y., Han Z., He K., Zhang Y., Wu D., Chen H. (2024). The effects of probiotics supplementation on *Helicobacter pylori* standard treatment: An umbrella review of systematic reviews with meta-analyses. Sci. Rep..

[9] Yap T.W.-C., Gan H.-M., Lee Y.-P., Leow A.H.-R., Azmi A.N., Francois F., Perez-Perez G.I., Loke M.-F., Goh K.-L., Vadivelu J. (2016). *Helicobacter pylori* Eradication Causes Perturbation of the Human Gut Microbiome in Young Adults. PLoS ONE.

[10] Hsu P.-I., Pan C.-Y., Kao J.Y., Tsay F.-W., Peng N.-J., Kao S.-S., Wang H.-M., Tsai T.-J., Wu D.-C., Chen C.-L. (2018). *Helicobacter pylori* eradication with bismuth quadruple therapy leads to dysbiosis of gut microbiota with an increased relative abundance of *Proteobacteria* and decreased relative abundances of *Bacteroidetes* and Actinobacteria. Helicobacter.

[11] Yanagi H., Tsuda A., Matsushima M., Takahashi S., Ozawa G., Koga Y., Takagi A. (2017). Changes in the gut microbiota composition and the plasma ghrelin level in patients with *Helicobacter pylori*-infected patients with eradication therapy. BMJ Open Gastroenterol..

[12] Hu Y., Zhang Z.-Y., Wang F., Zhuang K., Xu X., Liu D.-S., Fan H.-Z., Yang L., Jiang K., Zhang D.-K. (2025). Effects of amoxicillin dosage on cure rate, gut microbiota, and antibiotic resistome in vonoprazan and amoxicillin dual therapy for *Helicobacter pylori*: A multicentre, open-label, non-inferiority randomised controlled trial. Lancet Microbe.

[13] Liou J.-M., Chen C.-C., Chang C.-M., Fang Y.-J., Bair M.-J., Chen P.-Y., Chang C.-Y., Hsu Y.-C., Chen M.-J., Chen C.-C. (2019). Long-term changes of gut microbiota, antibiotic resistance, and metabolic parameters after *Helicobacter pylori* eradication: A multicentre, open-label, randomised trial. Lancet Infect. Dis..

[14] He C., Xie Y., Zhu Y., Zhuang K., Huo L., Yu Y., Guo Q., Shu X., Xiong Z., Zhang Z. (2022). Probiotics modulate gastrointestinal microbiota after *Helicobacter pylori* eradication: A multicenter randomized double-blind placebo-controlled trial. Front. Immunol..

[15] Tang B., Tang L., Huang C., Tian C., Chen L., He Z., Yang G., Zuo L., Zhao G., Liu E. (2021). The Effect of Probiotics Supplementation on Gut Microbiota After *Helicobacter pylori* Eradication: A Multicenter Randomized Controlled Trial. Infect. Dis. Ther..

[16] Oh B., Kim J.W., Kim B.S. (2016). Changes in the Functional Potential of the Gut Microbiome Following Probiotic Supplementation during *Helicobacter pylori* Treatment. Helicobacter.

[17] Wang L., Yao H., Tong T., Lau K., Leung S.Y., Ho J.W.K., Leung W.K. (2022). Dynamic changes in antibiotic resistance genes and gut microbiota after *Helicobacter pylori* eradication therapies. Helicobacter.

[18] Zhao M., Zhang Y., Liu S., Wang F., Zhang P. (2025). Eradication of *Helicobacter pylori* reshapes gut microbiota and facilitates the evolution of antimicrobial resistance through gene transfer and genomic mutations in the gut. BMC Microbiol..

[19] Liu Y., Wang S., Yang F., Chi W., Ding L., Liu T., Zhu F., Ji D., Zhou J., Fang Y. (2022). Antimicrobial resistance patterns and genetic elements associated with the antibiotic resistance of *Helicobacter pylori* strains from Shanghai. Gut Pathog..

[20] Shi X., Zhang J., Mo L., Shi J., Qin M., Huang X. (2019). Efficacy and safety of probiotics in eradicating *Helicobacter pylori*: A network meta-analysis. Medicine.

[21] Liu Y.-H., Zhang J., Li D.-H., Zhang Y.-P., Li J., Guo Q.-Q., Zhu X.-J., Shi Y.-Q. (2025). The impact of probiotics on *Helicobacter pylori* eradication with bismuth quadruple therapy: A systematic review and meta-analysis. Int. J. Antimicrob. Agents.

[22] McFarland L.V., Malfertheiner P., Huang Y., Wang L. (2015). Meta-analysis of single strain probiotics for the eradication of *Helicobacter pylori* and prevention of adverse events. World J. Meta-Anal..

[23] Viazis N., Argyriou K., Kotzampassi K., Christodoulou D.K., Apostolopoulos P., Georgopoulos S.D., Liatsos C., Giouleme O., Koustenis K., Veretanos C. (2022). A Four-Probiotics Regimen Combined with A Standard *Helicobacter pylori*-Eradication Treatment Reduces Side Effects and Increases Eradication Rates. Nutrients.

[24] Márquez C.M., Álvarez P.F., Delgado T.V., Laria L.C., Arias F.A., Álvarez A.C., Rodríguez B.J.G. (2022). Randomized, double-blind, placebo-controlled clinical trial on the usefulness of probiotic *Lactobacillus reuteri* in bismuth-containing quadruple eradication therapy for infection with *Helicobacter pylori*. Rev. Esp. Enferm. Dig..

[25] Du L., Chen B., Cheng F., Kim J., Kim J.J. (2024). Effects of *Helicobacter pylori* Therapy on Gut Microbiota: A Systematic Review and Meta-Analysis. Dig. Dis..

[26] Zheng P.X., Fang H.Y., Yang H.B., Tien N.Y., Wang M.C., Wu J.J. (2016). *Lactobacillus pentosus* strain LPS16 produces lactic acid, inhibiting multidrug-resistant *Helicobacter pylori*. J. Microbiol. Immunol. Infect..

[27] Boyanova L., Gergova G., Markovska R., Yordanov D., Mitov I. (2017). Bacteriocin-like inhibitory activities of seven *Lactobacillus delbrueckii* subsp. *bulgaricus* strains against antibiotic susceptible and resistant *Helicobacter pylori* strains. Lett. Appl. Microbiol..

[28] Rezaee P., Kermanshahi R.K., Falsafi T. (2019). Antibacterial activity of lactobacilli probiotics on clinical strains of *Helicobacter pylori*. Iran. J. Basic Med. Sci..

[29] Dash D., Mishra V., Panda M.K., Pathak S.K. (2025). Effects of *Lactobacillus* spp. on *Helicobacter pylori*: A Promising Frontier in the Era of Antibiotic Resistance. Probiotics Antimicrob. Proteins.

[30] Chen Y., Teng T., Su Y., Chen W.Z. (2024). The effect of supplementing with *Saccharomyces boulardii* on bismuth quadruple therapy for eradicating *Helicobacter pylori*: A systematic review and meta-analysis of randomized controlled trials. Front. Med..

[31] Zhou B.G., Chen L.X., Li B., Wan L.Y., Ai Y.W. (2019). *Saccharomyces boulardii* as an adjuvant therapy for *Helicobacter pylori* eradication: A systematic review and meta-analysis with trial sequential analysis. Helicobacter.

[32] Li M., Xie Y. (2025). Efficacy and safety of *Saccharomyces boulardii* as an adjuvant therapy for the eradication of *Helicobacter pylori*: A meta-analysis. Front. Cell. Infect. Microbiol..

[33] Jiang Y.Z., Ma K., Cui C., Li Z.Y., Wang X.Y. (2025). Effect of *Saccharomyces boulardii* supplementation to bismuth quadruple therapy on *Helicobacter pylori* eradication. BMC Gastroenterol..

[34] Zojaji H., Ghobakhlou M., Rajabalinia H., Ataei E., Sherafat S.J., Moghimi-Dehkordi B., Bahreiny R. (2013). The efficacy and safety of adding the probiotic *Saccharomyces boulardiito* standard triple therapy for eradication of *H. pylori*: A randomized controlled trial. Gastroenterol. Hepatol. Bed Bench.

[35] Cárdenas P.A., Garcés D., Prado-Vivar B., Flores N., Fornasini M., Cohen H., Salvador I., Cargua O., Baldeón M.E. (2020). Effect of *Saccharomyces boulardii* CNCM I-745 as complementary treatment of *Helicobacter pylori* infection on gut microbiome. Eur. J. Clin. Microbiol. Infect. Dis..

[36] Zhang Y., Lu B., Dong Y., Zhang Y., Du Q., Chen Y., Zhang Z. (2024). *Saccharomyces boulardii* combined with triple therapy alter the microbiota in the eradication of *Helicobacter pylori* infection. Sci. Rep..

[37] Keikha M., Kamali H. (2022). The impact of *Saccharomyces boulardii* adjuvant supplementation on alternation of gut microbiota after *H. pylori* eradication; A metagenomics analysis. Gene Rep..

[38] Cifuentes S.G., Prado M.B., Fornasini M., Cohen H., Baldeón M.E., Cárdenas P.A. (2022). *Saccharomyces boulardii* CNCM I-745 supplementation modifies the fecal resistome during *Helicobacter pylori* eradication therapy. Helicobacter.

[39] Pais P., Almeida V., Yılmaz M., Teixeira M.C. (2020). *Saccharomyces boulardii*: What Makes It Tick as Successful Probiotic?. J. Fungi.

[40] Sakarya S., Gunay N. (2014). *Saccharomyces boulardii* expresses neuraminidase activity selective for α2,3-linked sialic acid that decreases *Helicobacter pylori* adhesion to host cells. APMIS.

[41] Yu J., Lv Y.M., Yang P., Jiang Y.Z., Qin X.R., Wang X.Y. (2023). Safety and effectiveness of vonoprazan-based rescue therapy for *Helicobacter pylori* infection. World J. Gastroenterol..

